# Design and implementation of a clinical decision support tool for primary palliative Care for Emergency Medicine (PRIM-ER)

**DOI:** 10.1186/s12911-020-1021-7

**Published:** 2020-01-28

**Authors:** Audrey Tan, Mark Durbin, Frank R. Chung, Ada L. Rubin, Allison M. Cuthel, Jordan A. McQuilkin, Aram S. Modrek, Catherine Jamin, Nicholas Gavin, Devin Mann, Jordan L. Swartz, Jonathan S. Austrian, Paul A. Testa, Jacob D. Hill, Corita R. Grudzen, Group Authorship: Corita R. Grudzen on behalf of the PRIM-ER Clinical Informatics Advisory Board, Benjamin Abella, David Allard, Jonathan Austrian, M. Fernanda Bellolio, Michael Blum, Todd Burstain, Jeffrey Caterino, Julie Cooper, Bruce Darrow, Marie-Carmelle Elie, Ahmed Elsayem, John Frenzel, Howard Goldberg, Corita Grudzen, Iris Herrera, John Howell, Allen Hsaio, Eric Isaacs, Karen Jubanyik, Ken Kawamoto, Sangeeta Lamba, Troy Madsen, Joseph Miller, Kei Ouchi, Rajesh Patel, Rajiv Pramanik, Lynne Richardson, Milisa Rizer, Elizabeth Schoenfeld, Timothy Shiuh, Ashley Shreves, Robert Swor, Audrey Tan, Paul Testa, Arvind Venkat, Kendall Webb, Howard Weeks, Robert White, Decker Wyatt, Matthew Zimmie, Erin Zimny

**Affiliations:** 1 Ronald O. Perelman Department of Emergency Medicine, New York University School of Medicine, 227 East 30th Street, New York, NY 10016 USA; 2 NYU Langone Health, Medical Center Information Technology, New York, NY USA; 3 Department of Emergency Medicine, Columbia University Vagelos College of Physicians and Surgeons, New York, NY USA; 4 Department of Population Health, New York University School of Medicine, New York, NY USA

**Keywords:** Electronic health records, Clinical decision support, Palliative care, Emergency medicine, Quality improvement

## Abstract

**Background:**

The emergency department is a critical juncture in the trajectory of care of patients with serious, life-limiting illness. Implementation of a clinical decision support (CDS) tool automates identification of older adults who may benefit from palliative care instead of relying upon providers to identify such patients, thus improving quality of care by assisting providers with adhering to guidelines. The Primary Palliative Care for Emergency Medicine (PRIM-ER) study aims to optimize the use of the electronic health record by creating a CDS tool to identify high risk patients most likely to benefit from primary palliative care and provide point-of-care clinical recommendations.

**Methods:**

A clinical decision support tool entitled Emergency Department Supportive Care Clinical Decision Support (Support-ED) was developed as part of an institutionally-sponsored value based medicine initiative at the Ronald O. Perelman Department of Emergency Medicine at NYU Langone Health. A multidisciplinary approach was used to develop Support-ED including: a scoping review of ED palliative care screening tools; launch of a workgroup to identify patient screening criteria and appropriate referral services; initial design and usability testing via the standard System Usability Scale questionnaire, education of the ED workforce on the Support-ED background, purpose and use, and; creation of a dashboard for monitoring and feedback.

**Results:**

The scoping review identified the Palliative Care and Rapid Emergency Screening (P-CaRES) survey as a validated instrument in which to adapt and apply for the creation of the CDS tool. The multidisciplinary workshops identified two primary objectives of the CDS: to identify patients with indicators of serious life limiting illness, and to assist with referrals to services such as palliative care or social work. Additionally, the iterative design process yielded three specific patient scenarios that trigger a clinical alert to fire, including: 1) when an advance care planning document was present, 2) when a patient had a previous disposition to hospice, and 3) when historical and/or current clinical data points identify a serious life-limiting illness without an advance care planning document present. Monitoring and feedback indicated a need for several modifications to improve CDS functionality.

**Conclusions:**

CDS can be an effective tool in the implementation of primary palliative care quality improvement best practices. Health systems should thoughtfully consider tailoring their CDSs in order to adapt to their unique workflows and environments. The findings of this research can assist health systems in effectively integrating a primary palliative care CDS system seamlessly into their processes of care.

**Trial registration:**

ClinicalTrials.gov Identifier: NCT03424109. Registered 6 February 2018, Grant Number: AT009844–01.

## Background

The emergency department (ED) represents a critical decision point in the trajectory of care for patients with serious life-limiting illness, as three-quarters of these patients visit the ED in the 6 months before death [1]. These visits can indicate worsening clinical and functional status, which often occur in the setting of a breakdown in the coordination of care [2, 3]. A gap exists in the delivery of goal-concordant care, with steadily increasing intensive care admissions from the ED despite the fact that most patients with serious illness prefer to be home at the end of life [4, 5]. Equipping emergency providers with basic skills and competencies in palliative care, commonly termed primary palliative care, affords an opportunity to align care trajectory with patient goals. Providing palliative care in the ED has been demonstrated to improve quality of life, decrease intensive care unit admissions, decrease inpatient hospital length of stay, improve symptom management, and decrease cost [6–11]. However, referral to palliative care remains low, with 78.5% of emergency medicine (EM) providers reporting they refer patients with palliative care needs less than 10% of the time, and only 10.8% of providers feel they use effective methods to screen or refer patients to palliative care [12]. Emergency providers have previously identified time constraints and implementation logistics as the most challenging limitations to providing palliative care services in the ED. [13] Despite these challenges, interest in education, training, and delivery of palliative care in the ED continues to grow [13–15].

To optimally deliver primary palliative care in the ED, patients who could benefit from these services must be quickly, and accurately, identified. Existing tools to efficiently identify patients who may benefit from palliative care in high-acuity settings are currently limited to multi-tier screening tools which involve additional staffing to assist in patient identification, or rely heavily on emergency provider judgement [16–18]. With the pervasiveness of electronic health records (EHR), institutions can leverage electronic clinical decision support (CDS) to assist providers in identifying patients most likely to benefit from primary palliative care and provide point-of-care clinical recommendations [19, 20]. These CDS tools have dramatically evolved over the past 25 years to support diagnosis, treatment, care-coordination, and prevention [21].

Development of a palliative care CDS tool would assist in the sensitive, rapid, and efficient identification of adults who may benefit from palliative care, rather than relying on providers to identify such patients on an ad hoc basis. Additionally, these tools may improve quality of care by assisting with adherence to guidelines to reduce variance in provider practice. Specifically, they could provide targeted recommendations- such as consultation to multi-disciplinary palliative care teams and medication recommendations. As such, we sought to determine if it was feasible and usable to create a novel CDS tool adapted from an existing palliative care screening tool entitled P-CaRES. Furthermore, we aim to describe the design, implementation, and monitoring of a CDS tool as part of a National Institutes of Health (NIH)-funded pragmatic trial aimed at improving quality of care for patients with serious life-limiting illness in diverse ED environments that vary in specialty geriatric and palliative care capacity, geographic region, payer mix, and demographics.

## Methods

### Design and implementation

An Emergency Department Supportive Care CDS tool (Support-ED), was developed at the Ronald O. Perelman Department of Emergency Medicine at NYU Langone Health (NYULH). The system was developed as part of an institutionally sponsored Value-Based Management initiative and an NIH grant titled, “Primary Palliative Care for Emergency Medicine (PRIM-ER).” [22]To develop Support-ED, creators of the tool devised a multistep process to ensure a comprehensive and practical tool was implemented. These steps included: 1) a scoping review of existing ED palliative care screening tools; 2) creation of a multidisciplinary workgroup to identify patient screening criteria and appropriate referral services; 3) initial design and usability testing using the System Usability Scale (SUS) questionnaire; 4) education of the ED workforce on the Support-ED background, purpose and use, and 5) the creation of a dashboard to monitor frequency of alert firing and correlation with targeted actions. This study was approved by the New York University School of Medicine Institutional Review Board.

#### Scoping review

As an initial step, a scoping review of validated screening tools for unmet palliative care needs in the ED was conducted in March 2018 by author AT utilizing Pubmed. Based on the review, the Palliative Care and Rapid Emergency Screening (P-CaRES) was identified as the only screening tool to meet our search criteria and identify emergency patients with serious, life-limiting illness who could benefit from palliative care services [12]. P-CaRES consists of a two part screening process. The initial step screens for life-limiting conditions, including end-stage organ disease, advanced cancer, septic shock or multi-organ failure in elderly patients, or a high chance of accelerated death (e.g. cardiac arrest). The second step of the tool screens for functional decline, uncontrolled symptoms, caregiver distress, or provider gestalt regarding limited prognosis [10]. Other palliative care screening tools were excluded from consideration since they were not validated and/or were only tested at a single site. The P-CaRES framework was subsequently used to identify structured clinical data points within the EHR that could be used as triggers for a CDS tool.

#### Multidisciplinary workgroup

A workgroup comprised of seven individuals- one emergency/pallative care physician, two clinical operations physician leaders, an emergency medicine physician with information technology expertise, a nurse informaticist, a care manager and a social worker with expertise in the ED were assembled to participate in a think aloud methodology. The meeting objectives and tasks were to provide recommendations on; 1) screening criteria, 2) targeted recommendations including consultation to palliative care or social work services; and 3) design specifications such as how, when, and for whom an alert would be generated. Weekly meetings were conducted in-person in order to obtain valuable insights on the CDS creation process. Notes were taken during each of the nine in-person meetings and data was reviewed following each meeting to extract themes. When disagreements among the group occurred, group discussions were held until consensus was reached across all stakeholders.

##### Screening criteria

The P-CaRES screening criteria was modified and adapted by the workgroup to initially meet the specific needs of the NYULH workflow and population, with the ultimate goal of developing a tool that could be applied across the 35 diverse EDs enrolled in the PRIM-ER intervention. To identify patients who would benefit from palliative care, the workgroup systematically reviewed each of the components of the P-CaRES tool and identified structured clinical data points within the EHR that would serve as a surrogate within the Support-ED tool. For example, in place of “end stage renal disease”, a “Glomerular Filtration Rate (GFR) < 15 ml/min/m^2^” was selected which could be easily extracted from the EHR. The finalized criteria included historical data points that were surrogates for serious life-limiting illness, such as the presence of an advanced care planning document and critical lab values extracted from the current ED encounter. The aim for the selection of these criteria was to accurately identify serious illness and capture those patients who could benefit from primary palliative care interventions and referral to services while remaining specific enough to prevent over-firing and alert fatigue.

##### Targeted recommendations

Once Support-ED identified a qualifying patient, emergency providers received an alert to initiate a preliminary goals-of-care discussion and to consider a referral to the appropriate consult services. To determine if a referral was required, clinical questions modeled from the P-CaRES tool, such as measurements for worsening functional status, the presence of uncontrolled symptoms, or unclear goals of care, were asked of the providers. If the response was “yes”, the alert recommended a referral to palliative care service and/or social work. The ED social workers, who serve as the institutional liaisons with community hospice agencies, also received automatic notifications for any patients presenting with a history of prior hospice enrollment. The multidisciplinary workgroup based the referral system options on clinical practice scope and local capacity of the ED care team and referral services.

##### Design specifications

After establishing the clinical firing criteria and follow-up workflow, the workgroup used an iterative design process to construct the Support-ED framework. Primary design considerations included 1) interruptive vs. non-interruptive alerts, 2) alert timing and 3) alert audience.

1. *Interruptive* vs. *Non-Interruptive Alerts:* Considering the high pace and patient volume of the emergency department, the workgroup determined interruptive alerts would be more effective than non-interruptive alerts for providers. Interruptive alerts force emergency providers to temporarily pause their workflows to acknowledge the trigger. To prevent alert fatigue, specificity was emphasized over sensitivity to avoid over-firing. Non-interruptive alerts were employed for social workers and care managers given differences in their workflows.
2. *Alert Timing:* To ensure emergency providers received sufficient time to evaluate patients and analyze pertinent clinical data, the alert identifying patients with serious illness fired 1 hour after provider assignment. This timing allowed the emergency provider the opportunity to review the patient’s record before initiating a goals of care conversation. In contrast, the alert notifying emergency triage nurses of active advance care planning documentation for critically ill patients fired immediately upon chart opening so this information could be urgently relayed to the treating provider to affect care trajectory.
3. *Alert Audience:* Rather than solely targeting emergency providers, alerts were established for emergency nurses, social workers, and care managers as well. Each alert served a different purpose that aligned with the specific roles and practice scope of each personnel type. In addition to serving a clinical purpose, Support-ED promoted teamwork and a collaborative approach.

#### Usability testing

Prior to activating Support-ED within the NYULH EHR (PRIM-ER Pilot Site), think-aloud usability testing was conducted with a cohort of ED staff, including nurses, physicians, and clinical operations leadership between August and September 2018. During these sessions, testers explored multiple clinical scenarios within the EHR test environment that elicited different alerts. The scenarios included active advance care planning documents, active hospice, or the identification of patients with a possible serious illness that would fire an alert depending on the role of the user as either a provider, nurse, or social worker. Participants then verbalized any questions or issues they identified to the facilitators during these sessions. Upon completion of the scenarios, participants completed the standard System Usability Scale (SUS) questionnaire regarding their summative experiences [23, 24]. The SUS is a 10 item questionnaire with one of five responses that range from Strongly Agree to Strongly Disagree [24]. The SUS tests perceived usability and ease of use, as well as learnability [25, 26]. The final score is calculated by subtracting 1 from odd numbered questions, and for even numbered questions subtracting the value from 5. Sum of the scores is then multiplied by 2.5 for a final score [23]. SUS scores have a range of 0–100 and a score above a 68 is considered above average [24, 27]. Verbal feedback from these sessions was then presented to the workgroup and modifications were incorporated into the tool prior to launch.

#### Education of ED workforce

As part of PRIM-ER, evidence-based, multi-disciplinary primary palliative care education and simulation-based workshops are a major component of the study. All full-time emergency attendings and physician assistants completed an online didactic course on primary palliative care knowledge and skills in needs assessment and referral. This was supplemented by a simulation workshop in end-of-life communication carried out by a group of emergency physicians with expertise in palliative care. Similarly, all full-time emergency nurses completed an online nurse-focused didactic course on primary palliative care knowledge and skills. Further details regarding the PRIM-ER protocol can be found in the previously published study protocol paper [22]. Immediately following these sessions, education on Support-ED was provided including the purpose and specific workflows associated with each of the alerts.

#### Monitoring

The final component of PRIM-ER includes audit and feedback, which includes monitoring of the CDS tool. At NYULH, a clinical dashboard was created utilizing Tableau software (Version 2019.2.2) [28] to track the frequency in which each alert fires as well as the number of consults that are ordered as a result of each distinct alert at a departmental level. The dashboard also captures qualitative data through a comment text field that is prompted when a provider acknowledges that they are overriding one of the alerts and they are required to input their rationale. This dashboard is monitored on a weekly basis by PRIM-ER researchers (AT, JS) following “go-live” of Support-ED. Findings are disseminated to the multidisciplinary workgroup and ED leadership on a bi-weekly basis and informed future adaptations of the tool.

## Results

Based on feedback and notes directly obtained from the workgroup meetings, three alerts within the Support-ED tool were generated. To gain further feedback and buy-in, stakeholders including ED service chiefs, hospital leadership, and leadership of each of the affected service lines provided additional perspective and insight. The iterative design process yielded three distinct patient scenarios that trigger an alert to fire. For each distinct alert that was developed the triggering criteria, target provider, and response options are outlined in Table 1.

**Table 1:** Description of Three Alerts Within Support-ED Tool

Alert Name	Triggering Criteria	Target provider	Response options
Advance Care Planning Document Present	Active advanced care planning document + ESI 1 or 2	Nurse	Inform and acknowledge
	Active advance care planning document	Provider	Initiate a goals of care conversation. Consider ordering a social work or palliative care consult.
Hospice	Previous discharge disposition to hospice	Social Worker/Care Manager	Inform and acknowledge
		Provider	Consider ordering a social work or palliative care consult.
Serious Life-limiting Illness without Advance Care Planning Documentation	Surrogates for “serious life limiting illness” including historical data points (previous order for palliative care consult, previous order for “Do Not Resuscitate”) and data points from current ED counter (e.g. albumin< 2 g/dL, GFR < 15 ml/min/m^2^)	Provider	Initiate a goals of care conversation. Consider ordering a social work or palliative care consult.

*ESI* Emergency Severity Index- a clinical triage acuity scoring tool between 1 and 5 1 = “most severe/urgent.”

**Fig. 1:**
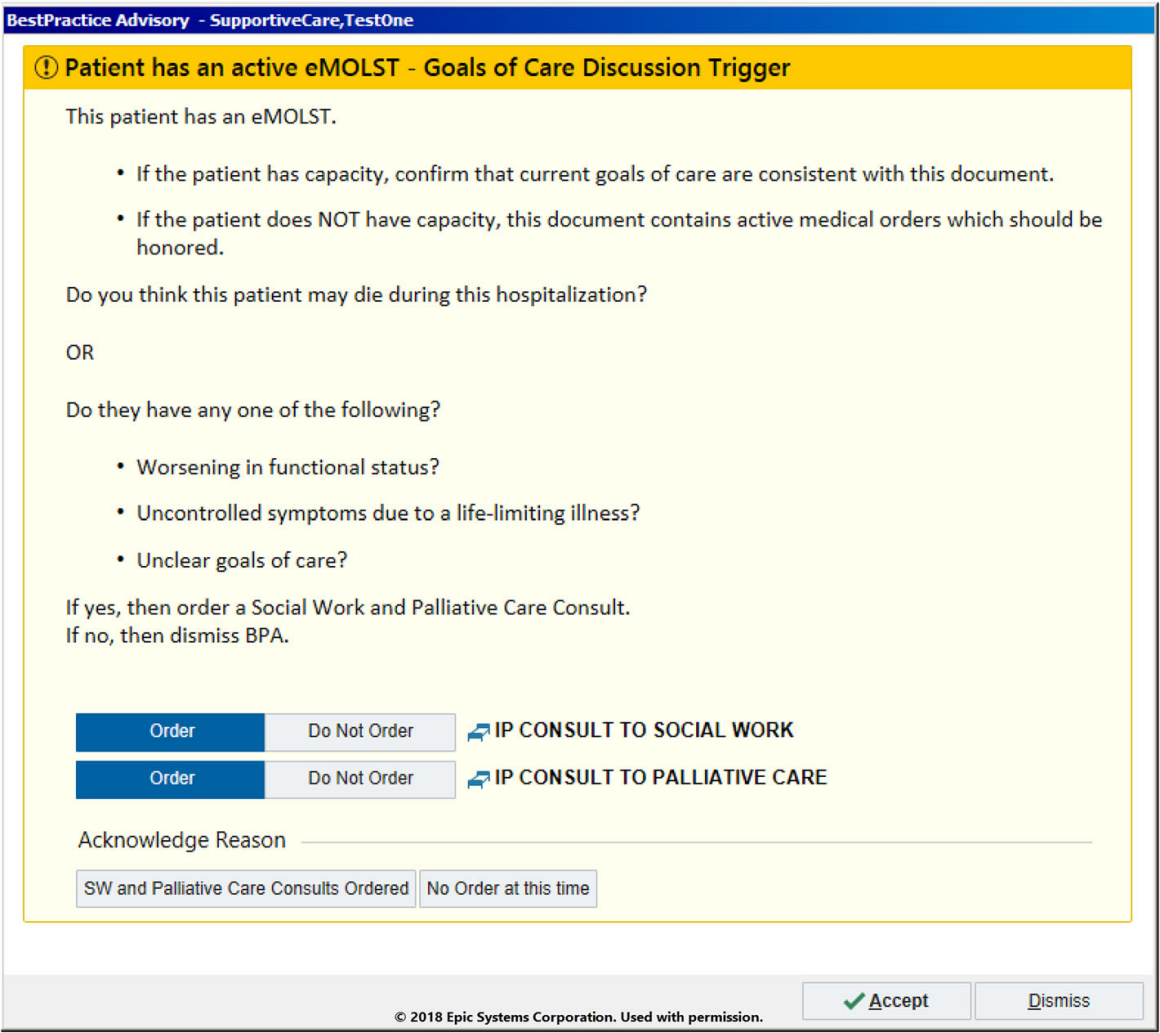
Example alert firing for a patient with an advanced care planning document on file

**Fig. 2:**
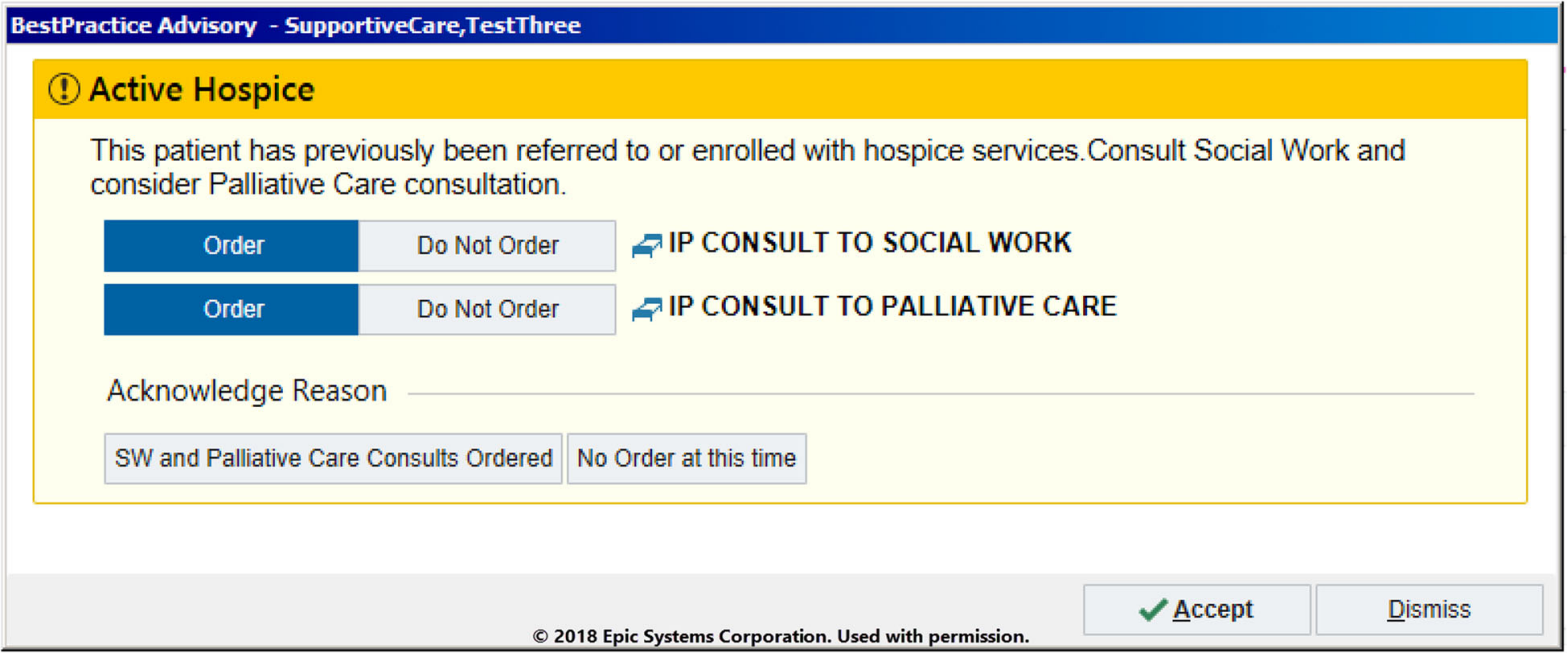
Example alert firing for a provider caring for a patient with an active hospice order

**Fig. 3:**
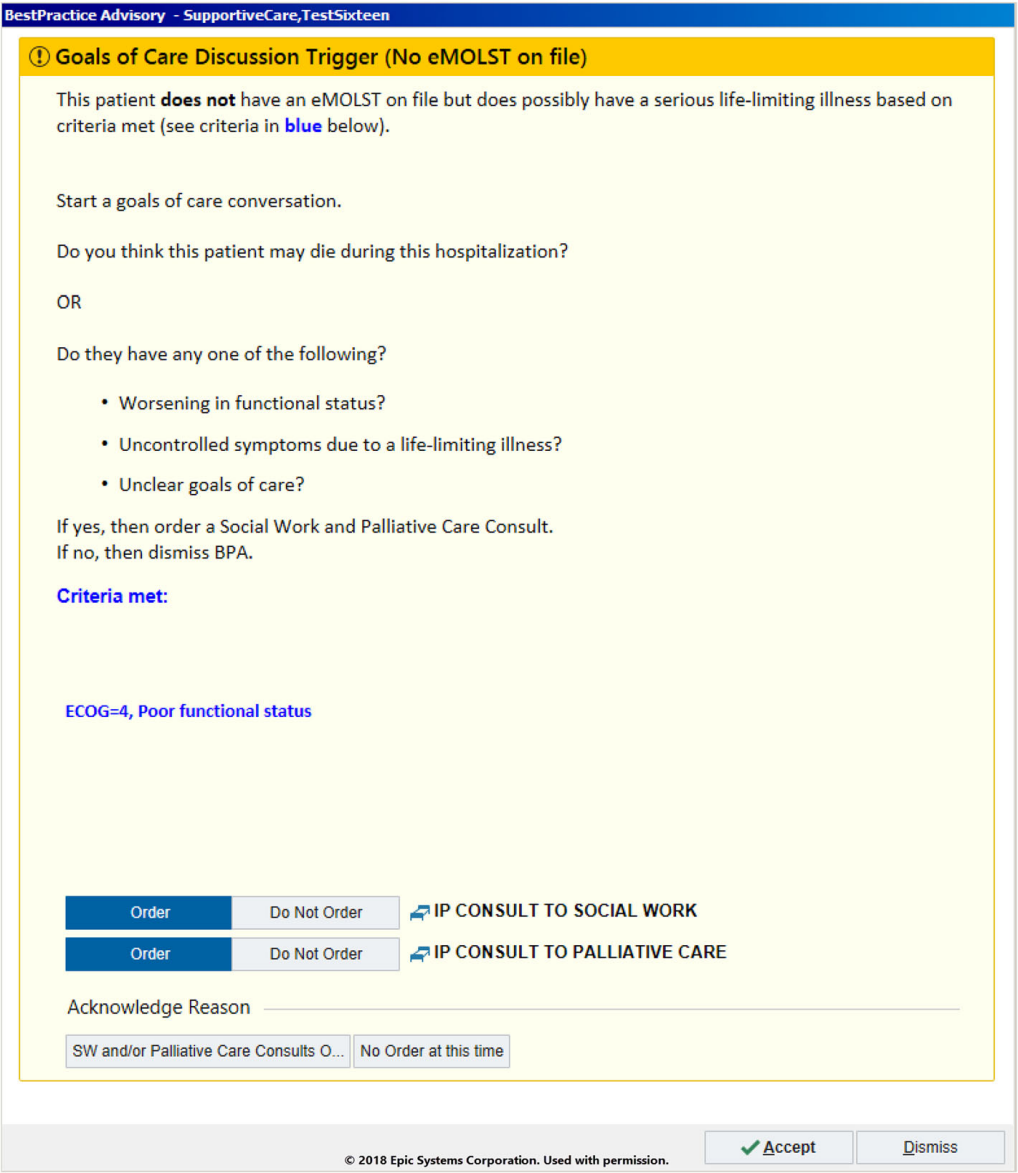
Example alert firing for a patient with a serious life-limiting illness without advance care planning documentation

Detailed description of each alert type in Table 1 is outlined below. Subsequently, screenshots from the EHR that demonstrate each specific alert are included in Figs. 1, 2, 3.

### Alert #1: advance care planning document present

The first alert is purely informational and is triggered by the presence of an advance care planning document within the EHR with an accompanying ESI (Emergency Severity Index- a clinical triage acuity scoring tool between 1 and 5 with 1 = “most severe/urgent.”) of 1 or 2 for the nurses and providers [29]. This alert targets nurses since they are typically the first to access a patient’s chart and could rapidly relay the information to a provider to ensure that the care provided would be aligned with the patient’s previously specified wishes. An alert (Fig. 1) subsequently fires for emergency providers recommending the initiation of a goals of care conversation and consideration of a palliative care and/or social work consult.

### Alert #2: hospice

The second alert (Fig. 2) is triggered by a previous discharge disposition to home or inpatient hospice. If the patient presented to the ED subsequent to the enrollment with hospice services, an informational alert fires for the providers. A similar alert also fires for the ED care managers and social workers to initiate care coordination, address social needs, and to contact the hospice agency.

### Alert #3: serious life-limiting illness without advance care planning documentation

The third alert (Fig. 3), designed to identify patients with serious life-limiting illnesses, is triggered by the historical data points and current encounter data points as show in Table 2. The presence of any of these data points triggers an alert for providers recommending the initiation of a goals-of-care conversation and consideration of a palliative care and/or social worker consult. In addition, to reduce immediate dismissals of the three alerts and to improve adherence, we required providers to manually input a reason for not following the advised action. This forced providers to pause and consider their decision before moving forward in overriding the alerts.

**Table 2:** Alert Criteria That Triggers Alert #3 in Support-ED

Historical Data Elements	
Mandatory surprise question: “Would you be surprised if this patient died within the previous 6 months?” (No) [30, 31]	
Previous palliative care consult	
Previous order for “Do Not Resuscitate”	
Last hospital disposition to a long-term acute care facility or nursing facility	
Previous scanned document of Consent to Withhold or Withdraw Life Sustaining Treatments	
Eastern Cooperative Oncology Group (ECOG) Score 3 or 4	
Current Encounter Data Elements	
Initiation of cardiac arrest documentation	
Active order for mechanical ventilation	
Active order for non-invasive ventilation	
GFR < 15 ml/min/m2	
Albumin < 2 g/dL	
Bicarbonate < 10 mEq/L	
pCO2 < 70 mmHg	

GFR Glomerular filtration rate.

pCO_2_ Partial pressure of Carbon Dioxide.

### Usability testing

Ten ED staff (physicians *n* = 7, nurses *n* = 3) completed the PRIM-ER SUS test on August 9, 2018. The users scored an average of 92.5 (75–100, SD = 7.56). A minimum average score of 85 is considered “excellent,” for perceived ease of use [16] Based on these results, the PRIM-ER research team felt confident in moving the CDS into the initial testing phase.

### Initial testing, implementation, and adaptations

To ascertain the frequency of the alerts firing, the Support-ED tool ran in the background while alerts to providers were silenced for 4 weeks in September 2018 prior to the formal launch or “go-live.” During this period, the three alerts together fired 844 times out of approximately 9000 total encounters across three clinical sites (9% of encounters).

During the initial 4-week period following launch of the tool, alert frequency was closely monitored, as well as correlations to consults ordered (Table 3). In addition, when the alert recommendation was not followed, qualitative feedback from providers was collected.

**Table 3:** Dashboard Monitoring Data of Support-ED Following Initial Launch

Alert	Provider Frequency: n	Percentage Receiving Consults (Palliative Care or Social Work)
Alert #1: Advance Care Planning Document Present	Nurse: 21	52.4%
	Emergency Provider^a^: 106	37.7%
Alert #2: Hospice	Social worker/Care manager: 17	41.2%
	Emergency Provider: 18	44.4%
Alert #3: Serious Life-Limiting Illness with No Advance Care Planning Document	Emergency Provider: 368	31.5%

^a^Emergency provider = emergency attendings, physician assistants

The qualitative data extracted from the dashboard revealed insights and rationales for why providers were overriding the alerts. Some examples included, but are not limited to, “will order pending family discussion,” “have not yet evaluated patient,” “patient acutely ill,” “ED patient without significant risk of death in ED,” “not a member of the primary care team,” and “I am seeing her as an outpt [outpatient] consultant after discharge.”

Within the override comments open text field, some providers used this area as an opportunity to express concerns. For example, one provider noted “please stop alerting me I just got here. I do not know if they need this yet. This is very disruptive to flow.” Additionally, another provider expressed that, “alarm fatigue is dangerous.” The dashboard monitoring of both the quantitative and qualitative data proved invaluable, as it was the impetus for improving the tool’s specificity and acceptability.

Based on the early data obtained from the dashboard, modifications were made rapidly to maximize buy-in and minimize alert fatigue and are described in Table 4.

**Table 4:** Adaptions to Support-ED Tool and Associated Rationale

Adaptation	Rationale
Alert #3 fires only for patients with an ESI of 1, 2 or 3	Resulted from provider feedback regarding the lack of utility of this firing on lower acuity patients.
If a palliative care consultation was already placed, Alert #3 does not fire	Amended to reduce the redundancy of orders.
Update all three alerts to fire for all the providers on the ED care team	Goal was to notify each of the providers on the ED care team instead of for example, only the attending provider.
Discontinue all three alerts from firing for providers that are not part of the ED care team (e.g. consultants)	Amended to target the right provider.
Update all three alerts to fire only once for each ED provider	Amended to reduce the redundancy of alert firing.
Firing of Alert #1 and Alert #3 changed from T + 60 min to T + 90 min after ED arrival	Based on provider feedback recommending firing later to allow sufficient time for patient evaluation and analysis of lab results.
Removal of “previous discharge disposition to nursing home” and “GFR < 15 ml/min/m2” from criteria for Alert #3	Based on dashboard feedback, these two criteria led to the most frequent firing and thus, these two were removed to increase alert specificity.
Suspension of Alert #3	Based on negative comments and over-firing, the decision was made to suspend this alert.

## Discussion

The Support-ED clinical decision support tool was developed to address a need for clinically relevant and timely identification of patients with palliative care needs in the ED coupled with actions relevant to care providers in this setting. Important lessons were learned from the development and initial launch of Support-ED at NYULH. Primarily, the modification of the P-CaRES screening survey into a functional CDS tool was scored highly for usability, as indicated by the SUS score of 92.5 (75–100, SD = 7.56) by NYULH ED providers. This is similar to the usability and acceptability testing of other ED palliative care screening tools. For example, 80.5% of emergency providers who tested the P-CaRES tool felt it would be useful for their practice [12], and 70% of providers indicated a content-validated palliative care screening tool developed by Ouchi et al. (2017) was acceptable [32]. In contrast, the percentage of encounters that identified palliative care needs from the CDS tool (9%) is much lower compared to the 32% positive screening found during feasibility testing of the Ouchi et al. tool. However, it is difficult to compare Support-ED with the Ouchi et al. tool since Support-ED is a real-time CDS tool, compared to a retrospective survey applied for the Ouchi et al. tool [32].

The importance of the dashboard for audit and feedback was critical to refining and monitoring our CDS tool. As suggested by Wright et al., the “importance of monitoring and evaluating decision support interventions after they are deployed and improving them continuously” cannot be overemphasized [33]. In our case, this information provided the workgroup with critical information that informed the amendments and modifications that were made to the tool. Despite the workgroup’s best efforts at modifying the “Serious Life-Limiting Illness with No Advance Care Planning Document” alert, feedback continued to be negative with persistent over-firing and thus, this alert was eventually suspended.

One important consideration that was subsequently deliberated by the workgroup was the targeted outcome for the alerts. A positive screen was initially defined as those alerts that generated a consultation to either palliative care or social work. Given the focus of the study interventions on providing the ED care team with the tools to improve primary palliative skills and to carry out goals of care conversations without relying on consultants, targeting this outcome primarily proved to be inaccurate. A subsequent quality improvement project focused on the creation of an advance care planning note specifically for emergency providers. Utilizing this as an additional outcome measure may more accurately capture the progress of emergency providers in carrying out these conversations.

Overall, there is a dearth of literature on CDS development and implementation in the context of palliative care, as well as integration in an ED setting [34]. To date, what is known is that CDS tools can play an integral role in assisting healthcare providers in providing patients optimal care by providing patient-specific recommendations at the point of need [11, 35]. Using the current body of literature on CDS challenges [36] and guidelines including best practices and principle guidelines outlined by Wright et al. [33] and the GUIDES checklist [37], we anticipated and developed strategies to overcome these challenges. Leveraging key stakeholders, aligning with organizational priorities and goals, and employing an iterative process for continual improvements and monitoring the usability led to our success [33].

### Limitations

There were several limitations we encountered during the design and implementation of this CDS tool. There is currently little evidence available in the literature describing which data elements should be utilized to identify patients with serious life-limiting illness within the EHR—thus we used expert consensus to determine which computer-interpretable data elements were utilized within the alerts. Second, CDS tools universally encounter barriers to acceptance and adoption by providers. Based on empirical studies and current recommendations, a major key to success for CDS tools is the integration into clinical workflow [35]. There is no standard for clinical workflow in the ED given the diversity of patient presentations and individual practice patterns making seamless integration of the alert into workflow challenging. This may have led to provider frustration and alert fatigue and as a result, less adherence to alert recommendations.

### Adaptation and future directions

To date (as of December 2019), the PRIM-ER research team has successfully aided in the implementation of a tailored Support-ED within the first 10 sites enrolled in the PRIM-ER intervention. Each site will deploy Support-ED in a stepped-wedge, randomized design [22]. To best tailor Support-ED to the unique workflows and environments of each participating site, interviews were conducted with key local stakeholders comprised of palliative care, emergency nursing, social work/case management, informatics, and ED operations representatives. These interviewers acquired information on health system palliative care resources, palliative care- related CDS tools currently utilized in the EHR, how to best customize the CDS to existing workflows, cultural norms that might alter receptivity to CDS, the CDS approval process, and current audit and feedback or quality metric reporting tools.

**Fig. 4:**
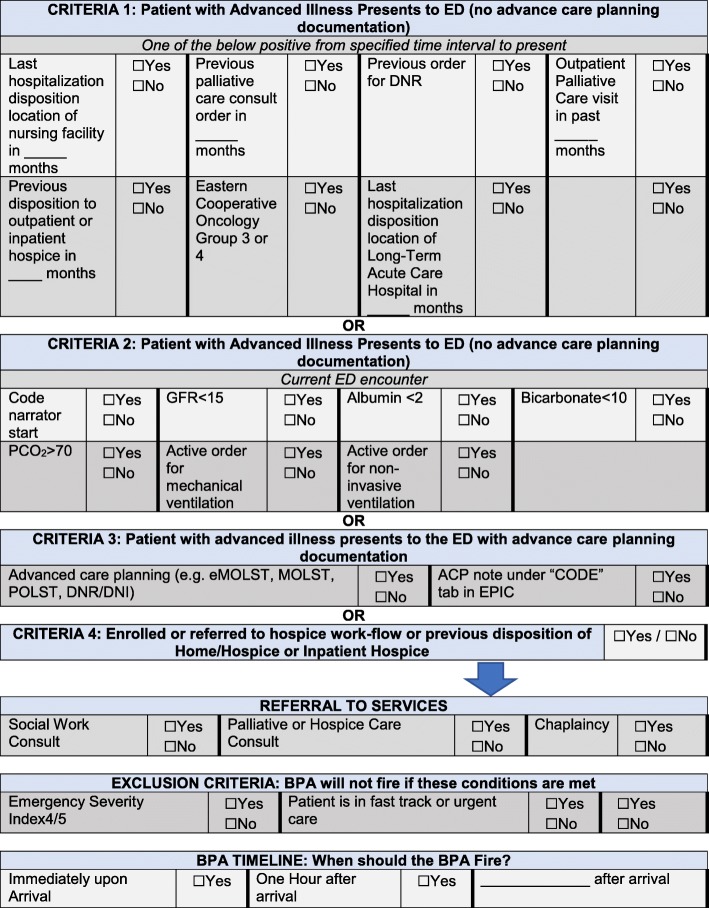
PRIM-ER clinical decision support streamlined mapping document

To adapt Support-ED to each health system’s unique workflows and best assimilate the feedback of all stakeholders, a CDS mapping document was created (Fig. 4). This modifiable document allows stakeholders to select, remove, and insert additional criteria to trigger the alerts and elicit specified outcomes. This document also will allow stakeholders to restructure and modify elements of the tool, such as the timing of the alerts firing or format of alerts to best serve their institutional needs. At each enrolled site, we gather written feedback from all stakeholders, and that feedback is compiled into a single mapping document and returned to each site for further refinement. From there, the NYULH team shares the baseline build with other institutions using the same EHR to provide sites with modifiable variables and template logic for ease of reproducibility and adaptation.

As not all sites utilize Epic, Support-ED is designed to be EHR-agnostic so that it can be generalizable to all sites within the intervention, as well as other sites not enrolled. For example, one of the study sites recently implemented a modified version of the Support-ED within Cerner's EHR, utilizing the NYULH build as a foundation to customize their CDS tool. Upon study completion at all 35 sites, the overall goal is to share Support-ED more broadly through proper dissemination strategies so other health systems can tailor and adapt the build to their EHRs.

## Conclusions

CDS alerts can be an effective tool in the implementation of primary palliative care quality improvement best practices. Health systems should thoughtfully consider tailoring and customizing their CDS tool in order to adapt to their unique workflow and environments. The findings of this research can assist health systems in the effective adaptation and seamless integration of a primary palliative care CDS tool into their standards of care.

## Data Availability

The datasets used and/or analysed during the current study are available from the corresponding author on reasonable request.

## Abbreviations

BPA: Best Practice Alert
CDS: Clinical Decision Support
ECOG: Eastern Cooperative Oncology Group
ED: Emergency Department
EHR: Electronic Health Record
EM: Emergency Medicine
ESI: Emergency Severity Index
GFR: Glomerular Filtration Rate
NIH: National Institutes of Health
NYULH: New York University Langone Health
P-CaRES: Palliative Care and rapid Emergency Screening
PRIM-ER: Primary Palliative Care for Emergency Medicine
Support-ED: Supportive Card Clinical Decision Support Bundle
SUS: System Usability Scale
